# Persistence of intraocular JC-virus associated with a MacTel phenotype

**DOI:** 10.1097/QAD.0000000000004240

**Published:** 2025-06-26

**Authors:** Nathan Hupin, Florence Hoogewoud, Ferenc B. Sallo, Sandra Vermeirsch, Veronique Suttels, Yan Guex-Crosier

**Affiliations:** a Department of Ophthalmology, Jules-Gonin University Eye Hospital, FAA, Lausanne, Switzerland; b VisIoNS, Institute of NeuroScience, Université Catholique de Louvain; c Department of Ophthalmology, Cliniques Universitaires Saint-Luc, Brussels, Belgium; d Department of Infectious Diseases, University Hospital of Lausanne, Lausanne, Switzerland.

## Introduction

The JC virus (JCV) is a polyomavirus known for establishing persistent, latent, and asymptomatic infections within the general population. Under conditions of compromised cellular immunity, notably in HIV infections, the virus can undergo reactivation and replication, leading to genomic rearrangements and acquisition of new tropisms, notably for the central nervous system where it can be responsible for progressive multifocal leukoencephalitis (PML) [1,2].

The clinical presentation of PML varies depending on the host's immunity, ranging from the classic, purely infectious to the immune recovery inflammatory syndrome (IRIS). The latter describes a clinical worsening associated with host immunity restoration after introduction of HAART [1]. The spectrum of neurological damage may present as ophthalmological complaints with the development of progressive retrochiasmal visual field defects [3], supranuclear and nuclear cranial nerve palsies, or nystagmus ataxia [4].

From an ocular perspective, immune recovery inflammatory syndromes (IRIS) have been described as immune recovery uveitis (IRU), induced by an immune response targeting intraocular viral antigens of viruses such as cytomegalovirus (CMV). This inflammation is characterized by vitritis, cystoid macular edema (CME), and epiretinal membrane [5].

Following immune reconstitution, a chronic and asymptomatic carrier state of JCV is known in the kidneys, bone marrow, lymphoid tissues and in the cerebrospinal fluid (CSF) of certain patients [6]. The presence of JCV in retinal tissue has been demonstrated by postmortem histological studies [7].

This report seeks to advance current knowledge on JCV pathogenicity by reporting its detection in-vivo in the eye of a patient with HIV infection, presenting with atypical bilateral macular changes years after a PML.

## Case report

We report the case of a 45-year-old man who presented to our accidents and emergencies (A&E) service of the University of Lausanne at baseline with a CDC stage C3 HIV infection. At the time of admission, his CD4^+^ cell count was 9 cells/μl, and his HIV-1 viral load was 150 000 copies/ml. He was diagnosed with pneumocystis pneumonia (PCP) and did not present neurological symptoms at the initial work-up. HAART was initiated (TAF/FTC-DTG-MVR adapted to the resistance profile of the patient's partner) and the viral load became undetectable (<50 copies/ml) 9 weeks after HAART initiation while the CD4^+^ cell count rose from 78 cells/μl (3.8%) to 119 cells/μl (7.1%). He exhibited right lateral hemianopsia 12 weeks after treatment initiation. Brain MRI revealed bilateral occipital lesions compatible with PML, unmasking IRIS. JCV PCR testing was performed on CSF alone, yielding a positive result of 347 copies/ml; testing was not conducted on other samples.

**Fig. 1:**
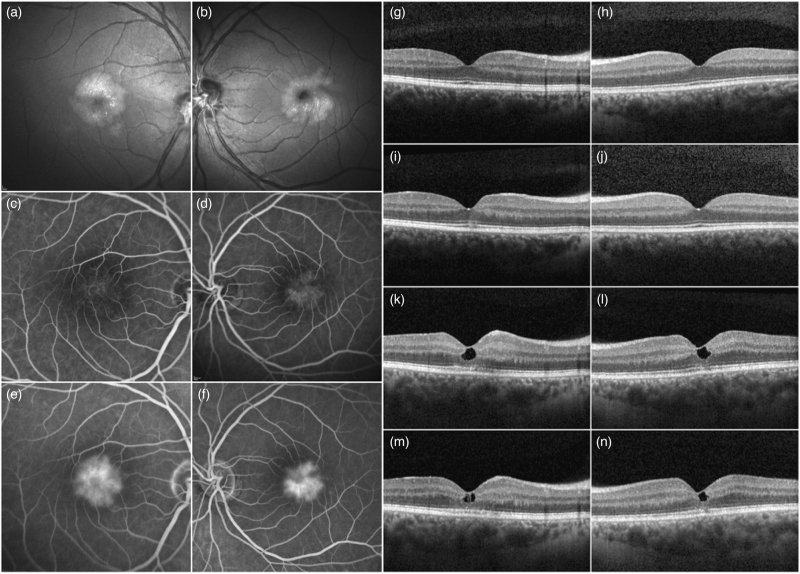
Blue light reflectance (BLR), fluorescein angiography (FA), and transfoveal OCT B-scan time series of the right and left eyes.

The ophthalmological examination at this time was unremarkable (Fig. 1g, h), except for the hemianopsia. The patient recovered from this episode and his HIV-VL remained undetectable, with a stable neurological status.

(a,b) BLR images of the right and left eyes. (c,d) Early phase FA (25 s) of the right and left eyes. (e,f) Late-phase (8 min) FA images of the right and left eyes. OCT images of the right and left eyes. (g,h) At baseline, (i,j) eleven months later, (k,l) three and a half years later. (m,n) Following treatment the overall retinal thickness decreased, with persisting hyperreflectivity and low reflective spaces, ONL thinning, EZ and IZ disorganization, and in the left eye a small EZ break.

Eleven months later, however, macular optical coherence tomography (OCT) of the right eye revealed a widening and blurring of the interdigitation and ellipsoid zones (IZ and EZ) and a hyperreflectivity within the outer nuclear layer (ONL) in both eyes (Fig. 1i, j), which progressed slightly over the next 5 months. The patient was asymptomatic.

Three and a half years later, the patient presented to our eye-hospital with a complaint of a progressive decline in bilateral visual acuity. Examinations revealed bilateral inner retinal cystoid spaces in the absence of vitritis. OCT images revealed a hyperreflectivity of the inner nuclear, inner plexiform, and ganglion cell layers (INL, IPL, and GCL), a hyperreflectivity and thinning of the ONL with the appearance of a low reflective space between the internal limiting membrane (ILM) and the ONL, a disorganization, widening, and attenuation of the ELM and the ellipsoid zone and a partial loss of the interdigitation zone (Fig. 1k, l). Blue light reflectance images showed a perifoveal area with increased reflectivity and superficial retinal crystals arranged along the nerve fiber bundles of the ganglion cells. The outer boundaries of these areas were somewhat irregular, especially in the left eye (Fig. 1a, b). Fluorescein angiography (FA) revealed dilated, more widely spaced perifoveal capillaries (Fig. 1c, d) and perifoveal leakage in the late phase (Fig. 1e, f). There was no other sign of intraocular inflammation. Investigations carried out initially failed to identify an etiology, with metabolic, infectious, drug toxicity, general inflammatory, and vascular causes coming up inconclusive. An aqueous sample revealed the presence of JCV at 752 copies/ml and the absence of any other viruses. A comprehensive radiological and neurological work-up showed no new radiological brain lesions at MRI and a negative JCV PCR on lumbar puncture.

## Discussion

We report the progressive development of macular changes in the presence of intraocular JCV after immune reconstitution. This could suggest JCV as a potential ocular pathogen and a novel agent responsible for ocular IRIS [5,8,9]. The eye could be a primary site of infection, or a secondary extension to the migration of the virus along the optic nerve, part of the virus-infected central nervous system. The progressive disorganization of outer retinal layers may highlight an insidious chronic inflammatory retinal damage, albeit these lesions are currently not encompassed within the definition of IRIS. The combination of telangiectatic capillaries temporal to the fovea, a hyperreflectivity to blue light, superficial retinal crystals, and a loss of luteal pigment in a characteristic pattern are suggestive of type 2 macular telangiectasia (MacTel) [10]. However, the central retinal thickening, the OCT hyperreflectivity, and the extensive FA leakage are not typical for MacTel. The most likely explanation is the presence of dual pathologies, the JCV infection modulating the MacTel phenotype. At this point however, it cannot be excluded that the JCV may mimic the MacTel phenotype. Furthermore, a JCV infection may accelerate the progression of the signs of MacTel type 2.

## Conclusion

This report highlights the eye as a new site of invasion of the JCV. Ocular involvement occurred in the absence of active neurological involvement and in the absence of virus in the CSF. It remains to be determined if the presence of the virus is pathogenic on its own or if the eye could also be considered as a reservoir tissue of the virus. Systematic inclusion of JCV in anterior chamber tap of patients presenting with PML and macular edema could confirm our hypothesis.

## Acknowledgements

This work was collected through the online JIR-cohort system with the support of the AURIS Foundation, W. & E. Grand dʼHauteville Foundation, Ingvar Kamprad Fund, Fleurette Wagemakers Foundation, Kononchuk Family grant, Blatter Family grant, and Rhumatismes-Enfants-Suisse Foundation. Open access funding provided by University of Lausanne.

### Conflicts of interest

There are no conflicts of interest.
